# Aspirin Breaks the Crosstalk between 3T3-L1 Adipocytes and 4T1 Breast Cancer Cells by Regulating Cytokine Production

**DOI:** 10.1371/journal.pone.0147161

**Published:** 2016-01-21

**Authors:** Chia-Chien Hsieh, Yu-Shan Huang

**Affiliations:** Department of Human Development and Family Studies, National Taiwan Normal University, Taipei, Taiwan; University of South Alabama, UNITED STATES

## Abstract

Breast cancer is one of the most common cancers in women worldwide. The obesity process is normally accompanied by chronic, low-grade inflammation. Infiltration by inflammatory cytokines and immune cells provides a favorable microenvironment for tumor growth, migration, and metastasis. Epidemiological evidence has shown that aspirin is an effective agent against several types of cancer. The aim of this study is to investigate the anti-inflammatory and anti-cancer effects of aspirin on 3T3-L1 adipocytes, 4T1 murine breast cancer cells, and their crosstalk. The results showed that aspirin treatment inhibited differentiation and lipid accumulation by 3T3-L1 preadipocytes, and decreased the secretion of the inflammatory adipokine MCP-1 after stimulation with tumor necrosis factor (TNF)-α or conditioned medium from RAW264.7 cells. In 4T1 cells, treatment with aspirin decreased cell viability and migration, possibly by suppressing MCP-1 and VEGF secretion. Subsequently, culture of 4T1 cells in 3T3-L1 adipocyte-conditioned medium (Ad-CM) and co-culture of 3T3-L1 and 4T1 cells using a transwell plate were performed to clarify the relationship between these two cell lines. Aspirin exerted its inhibitory effects in the transwell co-culture system, as well as the conditioned-medium model. Aspirin treatment significantly inhibited the proliferation of 4T1 cells, and decreased the production of MCP-1 and PAI-1 in both the Ad-CM model and co-culture system. Aspirin inhibited inflammatory MCP-1 adipokine production by 3T3-L1 adipocytes and the cell growth and migration of 4T1 cells. It also broke the crosstalk between these two cell lines, possibly contributing to its chemopreventive properties in breast cancer. This is the first report that aspirin’s chemopreventive activity supports the potential application in auxiliary therapy against obesity-related breast cancer development.

## Introduction

In 2014, the World Health Organization estimated that over 600 million adults worldwide were overweight, comprising about 39% of adults, and 13% were obese, with 42 million children also being overweight or obese [1]. In obesity, the hyper- accumulation of adipose tissue is characterized by an accompanying low-grade inflammation. During the active stage, adipose tissue is associated with the increased infiltration of various types of immune cells, which secrete a series of pro-inflammatory adipokines and cytokines such as leptin, interleukin (IL)-6, IL-8, and tumor necrosis factor (TNF)-α [2–4].

The prevalence of obesity is related to increased risk and the progression of many cancers such as breast, colon, endometrial, esophageal, and renal cancer [5]. Interestingly, obesity is one of the major factors that have shown a consistent and strong link to the increased risk of breast cancer [5,6]. Breast cancer is now the most common cancer in women worldwide, with a prevalence about 4.3 million cases, accounting for 25% of all cancers in women, and a mortality rate of 1.29 million cases per year [7,8].

Cancer has complex effects on the immune system, influencing both innate and acquired immunity and involving inflammatory responses [9]. Chronic inflammation participates in the development of about 15–20% of malignancies worldwide and this has been revealed by epidemiological, experimental, and clinical studies [10]. Persistent inflammatory cell recruitment generates reactive oxygen species (ROS) and pro-inflammatory mediators that contribute to neoplastic transformation, resulting in tumor invasion and metastasis [11]. The tumor periphery consists of a variable combination of tumor cells, stromal fibroblasts, endothelial cells, and infiltrating leukocytes and macrophages [9]. Multiple biologically active molecules are secreted, such as macrophage chemoattractant protein-1 (MCP-1), vascular endothelial growth factor (VEGF), and plasminogen activator inhibitor-1 (PAI-1), contributing to angiogenesis involving cell proliferation, migration, and the remodeling of endothelial cells [12], and providing a microenvironment favorable for tumor growth.

Aspirin (acetylsalicylic acid) was synthesized in 1897, and was included in the non-steroidal anti-inflammatory drugs (NSAIDs) commonly used for relieving the symptoms of inflammation and protecting against coronary heart disease [13,14]. In the past few decades, accumulating epidemiological evidence has suggested a promising chemopreventive role for aspirin against various cancers [13,15]. Studies have revealed that aspirin reduces the inflammation associated with several types of cancer, such as colorectal, breast, lung, prostate, esophageal, stomach, and ovarian cancers [13]. Aspirin is an inhibitor of cycloxygenase (COX)-2, thereby implying its use as a potential chemopreventive agent in breast cancer, which is accompanied by overexpression of COX-2 [16].

A large and diverse body of epidemiological evidence has been gathered from human studies on the potential chemopreventive effects of aspirin use against breast cancer [15,17,18]. Recently, animal studies have also been conducted, which support breast cancer reduction with aspirin treatment [19,20]. However, the gastrointestinal side effects, optimal doses, duration, and combination with other compounds should first be clarified for the use of aspirin therapy in cancer prophylaxis. Whether its benefits outweigh the risks is still an undecided issue.

There are still few data exploring the chemoprevention of obesity-related breast cancer development. In this study, we investigated the anti-inflammatory and anti-cancer properties of aspirin on 3T3-L1 adipocytes, 4T1 murine breast cancer cells, and their crosstalk. A better understanding of the mechanistic links between obesity and breast cancer is crucially needed to clarify the targets and strategies required to mitigate the pro-cancer effects of obesity.

## Materials and Methods

### Cell culture and reagents

Mouse 3T3-L1 fibroblast and RAW264.7 macrophage were kindly provided by Dr. Lin of the National Taiwan University and Dr. Tsai of the National Taiwan Normal University (Taipei, Taiwan), respectively. The original cell lines were purchased from Bioresource Collection and Research Center, Food Industry Research and Development Institute (Hsinchu, Taiwan). Mouse 3T3-L1 and RAW264.7 cells were cultured in Dulbecco's modified Eagle's medium (DMEM, Caisson, Smithfield, UT, USA) containing 10% heat-inactivated calf serum (BS, Gibco, Grand Island, NY, USA) and fetal bovine serum (FBS, Genedirex, Las Vegas, NV, USA), respectively. Murine 4T1 breast cancer cells were purchased from American Type Culture Collection (ATCC, Manassas, VA, USA) and maintained in DMEM containing 10% heat-inactivated FBS with 1% penicillin/streptomycin/amphotericin B (Caisson) at 37°C in an incubator containing a humidified atmosphere of 5% CO_2_. Aspirin was purchased from Sigma (St. Louis, MO, USA), dissolved in dimethyl sulfoxide (DMSO, Sigma) as a stock solution, and then stored at −20°C. The concentration of DMSO in the vehicle group was equal to the 0.25% DMSO in the highest (5 mM) dose of aspirin.

### Differentiation of 3T3-L1 cells and quantification of lipid accumulation

*Differentiation of 3T3-L1 preadipocytes into adipocytes*. The 3T3-L1 preadipocytes were seeded at a density of 3 × 10^4^ cells/well in 24-well plates (Becton Dickinson, Franklin Lakes, NJ, USA) in DMEM supplemented with 10% FBS. After reaching full confluence over 2 days, the cells were induced to differentiate (designated as day 0) using medium I: DMEM containing 25 mM glucose, 0.5 mM 3-isobutyl-1-methylxanthine (Sigma), 0.2 μM dexamethasone (Sigma), 10 μg/ml insulin (Sigma), and 10% FBS for 4 days, and this was then changed to maintenance medium II: DMEM containing 25 mM glucose, 10 μg/ml insulin, and 10% FBS. During this stage, medium II was replaced every 3 days until 13 days after the initiation of the differentiation protocol to promote the maturation of adipocytes. The mature adipocytes were ready for further analysis.

*Quantifying lipid accumulation*. Different concentrations of aspirin at 0.5, 2, and 5 mM were used to treat 3T3-L1 cells during the differentiation period, after which the accumulated lipid droplets were visualized microscopically by staining cultured cells with Oil Red O (Sigma). Briefly, cells were washed with PBS and fixed using 10% paraformaldehyde at room temperature for 30 min, and then stained with Oil Red O solution for 40 min. The cultured cells were visualized under a microscope at 200× magnification (Nikon, Tokyo, Japan). The Oil red O dye was solubilized in 2-propanol and quantified by reading the absorbance at 500 nm using a microplate reader (BioTek, Winooski, VT, USA). The results were expressed as a percentage of the control, which was considered to be 100%. The relative lipid accumulation was calculated according to the formula (A_sample_ − A_blank_)/(A_control_ − A_blank_) ×100.

### Cell viability assay

The 3T3-L1 and 4T1 cells were seeded at a density of 3 × 10^4^ cells/well and 1 × 10^3^ cells/well, respectively, into 96-well plates (Becton Dickinson) and treated with various concentrations of aspirin at 0.1~10 mM for 24, 48, and 72 h. At the end of the treatment, cells were incubated in 0.5 mg/ml methylthiazole tetrazolium (MTT, Sigma) solution for 2 h at 37°C. The supernatant was aspirated and MTT formazan crystals were solubilized in DMSO for 15 min. The spectrophotometric absorbance at 540 nm was measured using a microplate reader (BioTek). The results were expressed as a percentage of the control, which was considered to be 100%. The relative cell viability was calculated according to the formula (A_sample_ − A_blank_)/(A_control_ − A_blank_) × 100.

### TNF-α and RAW-CM-induced adipocyte inflammation

*TNF-α induced adipocyte inflammation*. On day 12, 3T3-L1 adipocytes were treated with 0.5, 2, and 5 mM of aspirin and stimulated by 2.5 ng/ml TNF-α (PeproTech, Rocky Hill, NJ, USA) in DMEM containing 1% BS for 24 h. Cultured supernatants were collected and MCP-1 levels were measured by ELISA, according to the manufacturer’s instructions (R&D, Minneapolis, MN, USA).

*Preparation of conditioned medium from RAW 264*.*7 cells (RAW-CM)*. RAW 264.7 cells were plated in 24-well plates at a density of 5 × 10^5^ cells/well in DMEM containing 10% FBS and allowed to attach overnight. Cells were stimulated with lipopolysaccharide (LPS, 100 ng/ml, Sigma) for 24 h in DMEM containing 1% FBS, and the supernatants were collected, centrifuged at 1500 rpm for 5 min to remove cell debris, and used as RAW-CM. The 3T3-L1 cells were cultured in RAW-CM and treated with 0.5, 2, and 5 mM of aspirin for 24 h. The supernatants were collected and stored at −70°C for cytokine assays.

### Cell migration assay

Migration of 4T1 cells was assessed using the wound-healing assay. The 4T1 cells were plated at a density of 5 × 10^4^ cells/well in 48-well plates with 10% FBS/DMEM until they reached 80% confluence. The cell monolayer was carefully scratched using a 20 μL pipette tip, and then the medium was replaced with the aspirin treatment at 2, 5, and 10 mM. Images were captured using a microscope at 100× magnification with a camera (WS500, Whited, Taoyuan, Taiwan) at 0, 16, and 24 h. This assay was performed at least 3 times.

### Determination of MCP-1, PAI-1, and VEGF production by ELISA

The 3T3-L1 cells were seeded at a density of 3 × 10^4^ cells/24-well plate for differentiation into adipocytes. Cells were then treated with aspirin and stimulated for 24 h using TNF-α or RAW-CM. The 4T1 cells were seeded at a density of 1.5 × 10^5^ cells/48-well plate overnight. Cells were then treated with 2 and 5 mM aspirin for 24 or 48 h, respectively. Cultured supernatants were collected and cytokines such as macrophage chemoattractant protein (MCP-1), vascular endothelial growth factor (VEGF), and plasminogen activator inhibitor-1 (PAI-1) were analyzed by ELISA, according to the manufacturer’s instructions (R&D). Briefly, ELISA plates were coated with capture antibodies overnight at room temperature. The plates were washed and blocked for 1 h. After washing, the supernatant then being added and incubated at room temperature for 2 h. Then, the plates were washed before adding detection antibodies, horseradish peroxidase-conjugated streptavidin, and then substrate solution. Finally, the stop solution was added, and the absorbance was measured at 540 nm using a microplate reader (BioTek). The data was calculated according to the cytokine standard curve.

### Culture of 4T1 cells in adipocyte-conditioned medium

*Generation of adipocyte-conditioned medium (Ad-CM)*. When 3T3-L1 adipocytes were differentiated to day 12, the medium was replaced with 1% FBS/DMEM for 24 h. The supernatants were collected and used as adipocyte-conditioned medium (Ad-CM) for subsequent *in vitro* studies.

*Culture of 4T1 cells in Ad-CM*. To test the effect of Ad-CM on the growth of 4T1 cells, different concentrations of Ad-CM were prepared in fresh 1% FBS/DMEM medium with or without aspirin and then Ad-CM was used to culture 4T1 cells for 48 h. The CM contained 1% FBS in this study to provide a baseline level of nutrients needed for cellular function. The 4T1 cells were trypsinized and the cells numbers were counted using a Nucleocounter NC-3000 (ChemoMetec, Allerød, Denmark), according to the instructions of the manufacturer. Images were captured using a microscope with a camera WS500 (Whited) at 100× magnification.

*The effect of aspirin on cytokine production of 4T1 cells cultured in Ad-CM*. The 4T1 cells were seeded at a density of 4 × 10^4^/well in 48-well plates and treated for 48 h with 2 and 5 mM of aspirin prepared in half Ad-CM and half 1% FBS/DMEM medium to mimic the environment of breast cancer cells near adipocytes. The supernatants were collected and stored at −20°C for cytokine assays.

### Co-culture of 3T3 adipocytes with 4T1 breast cancer cells

To mimic the physiological environment of obesity-related breast cancer, the co-culture of tumor cells and adipocytes was carried out using a transwell culture system. The 3T3-L1 cells were seeded in 24-well plates and differentiated into adipocytes until day 13. The 4T1 cells were seeded in the upper chamber of a 24-well transwell culture system (0.4 μm pore size, Nunc, Waltham, MA, USA) at a density of 5 × 10^3^ cells/well. This co-culture system was maintained using 10% FBS/DMEM and treated with 2 and 5 mM of aspirin for 48 h. The 4T1 cells were trypsinized and the cells were counted using a Nucleocounter NC-3000 (ChemoMetec), according to the manufacturer's instructions. Cultured supernatants were collected and stored at −20°C for cytokines assays.

### Statistical analysis

All data were analyzed from three independent experiments, and data are presented as means ± standard error of the mean (SEM). Differences between the groups were analyzed by one-way ANOVA with the Least Significant Difference (LSD) post hoc test (IBM Statistical Product and Service Solutions (SPSS), version 19). A p value of less than 0.05 was considered statistically significant.

## Results

### Aspirin inhibited preadipocytes differentiation and adipose accumulation

**Fig 1:**
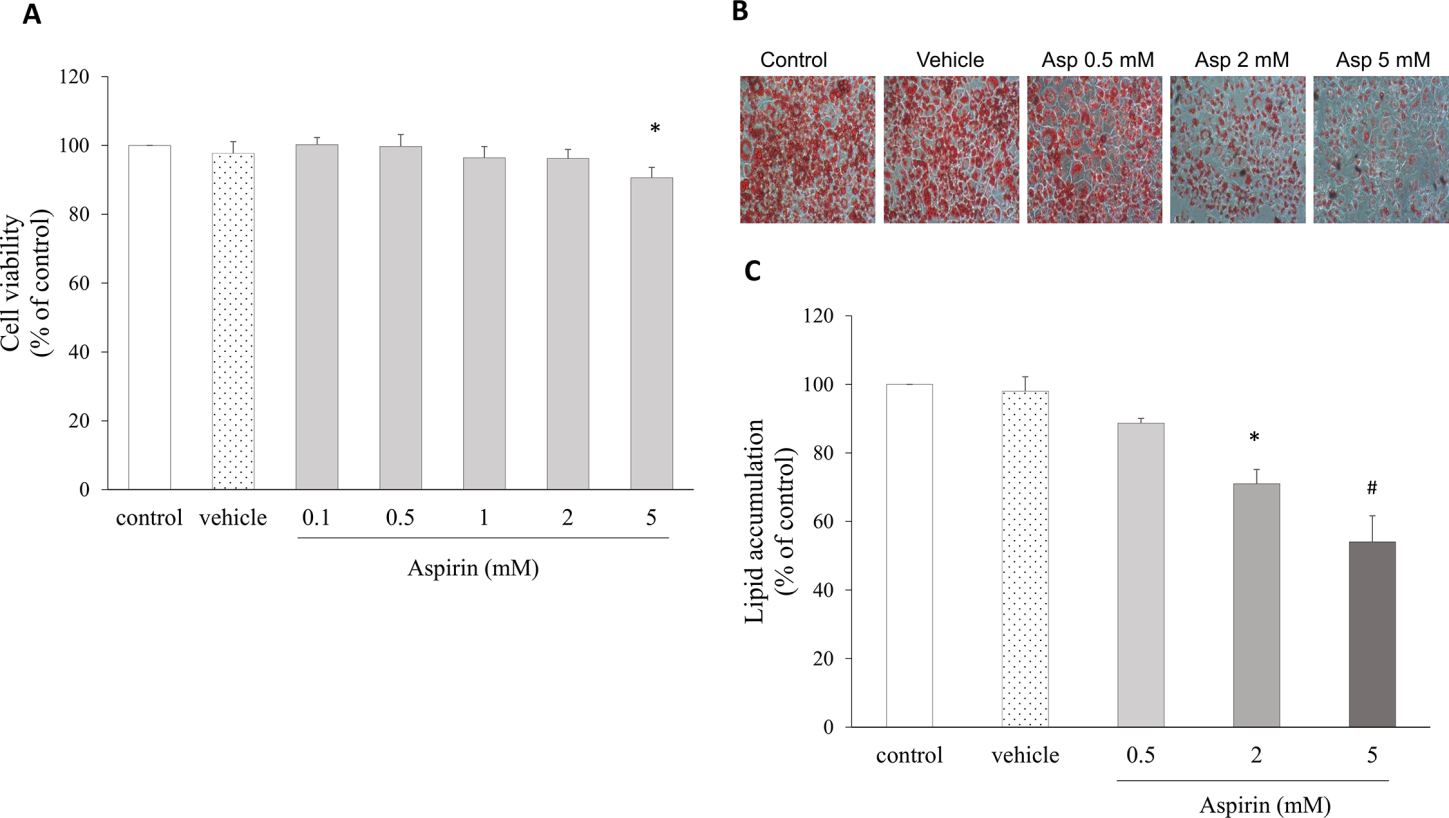
Aspirin inhibited preadipocyte differentiation and fat storage in mature 3T3-L1 adipocytes. **A.** Cell proliferation of 3T3-L1 cells treated with increasing doses of aspirin for 48 h, assessed by MTT assay. **B.** Cell morphology of oil accumulation in 3T3-L1 adipocytes treated with aspirin during preadipocyte differentiation; mature cells were stained with Oil Red O and observed microscopically at 200× magnification. **C.** The oil accumulated in 3T3-L1 cells was dissolved in 2-propanol and quantified by reading the absorbance at 500 nm using a microplate reader. Data are presented as mean ± SEM. Statistical analysis was done by one-way ANOVA and LSD post hoc test; significantly different at * p < 0.05 or # p < 0.001 vs. control group.

To investigate whether aspirin has effects on cell viability or the accumulation of lipid during the differentiation process in cultured adipocytes, 3T3-L1 cells were treated with concentrations of aspirin ranged from 0.1 to 5 mM. At the end of differentiation, the cells exhibited the adipocyte phenotype. The viability of cells treated with the highest dose (5 mM) of aspirin was significantly suppressed, but lower doses did not affect cell growth (Fig 1A). Cell morphology showed that aspirin decreased the percentage of 3T3-L1 cells stained with Oil Red O (Fig 1B) and suppressed lipid accumulation in a dose-dependent manner, with decreases of 11%, 29% (p = 0.001) and 45% (p < 0.001), for the 0.5, 2, and 5 mM treatments, respectively, compared with the control group (Fig 1C).

### Aspirin inhibited pro-inflammatory adipokine secretion in 3T3-L1 adipocytes

**Fig 2:**
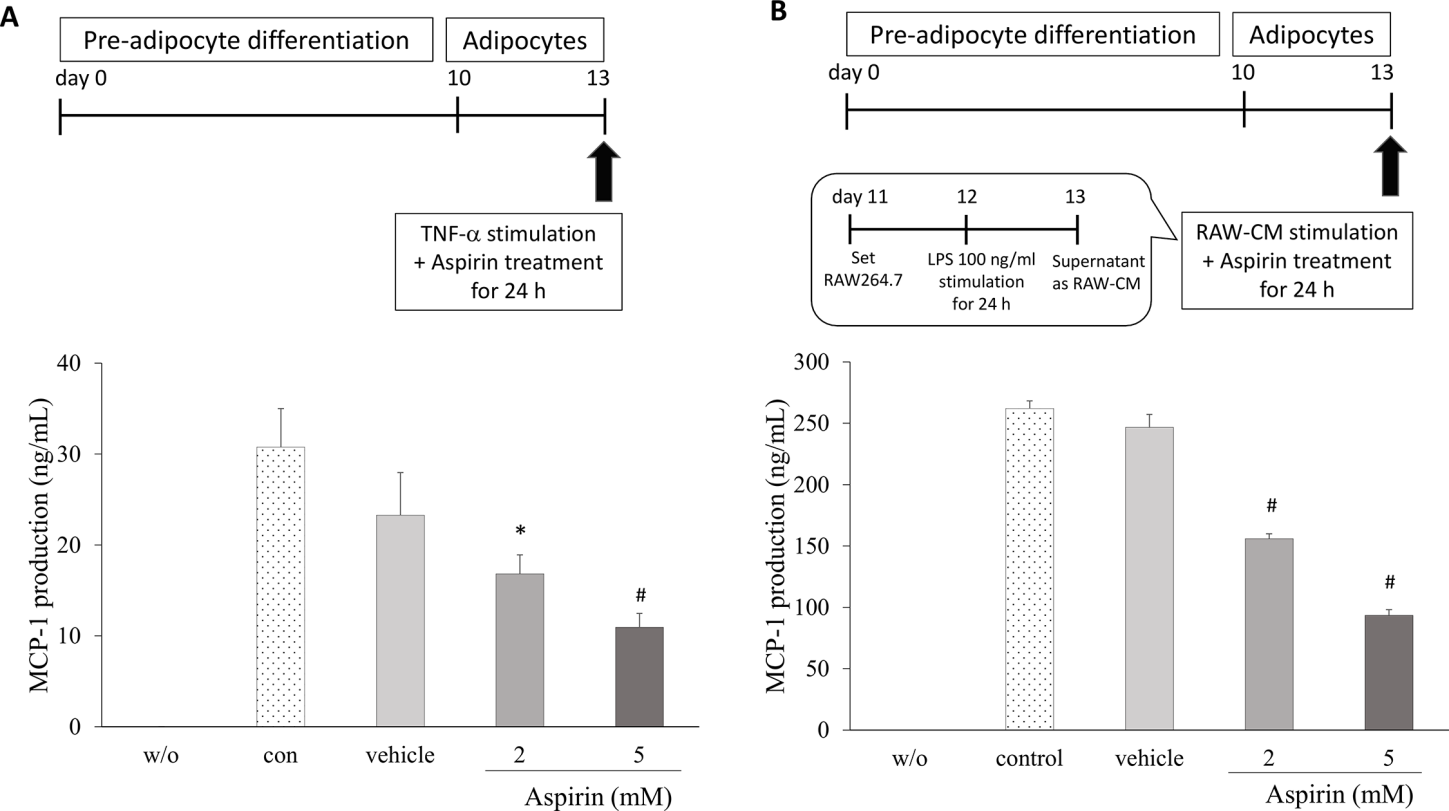
The effect of aspirin on MCP-1 secretion by 3T3-L1 adipocytes. **A.** The 3T3-L1 adipocytes were cultured in the presence or absence of aspirin and stimulated by 2.5 ng/mL TNF-α for 24 h. **B.** The 3T3-L1 adipocytes were cultured in the presence or absence of aspirin in RAW264.7 conditioned medium for 24 h. Data are presented as mean ± SEM. Statistical analysis was done by one-way ANOVA and LSD post hoc test; significantly different at * p < 0.05 or # p < 0.001 vs. control group.

To evaluate the anti-inflammatory actions of aspirin on 3T3-L1 adipocytes, the effects on inflammatory mediators were analyzed. The 3T3-L1 cells were treated with a series of aspirin concentrations, and stimulated by TNF-α to induce an inflammatory reaction. MCP-1 production apparently increased compared with unstimulated cells. Aspirin treatments showed a dose-dependent decrease by 18% and 46% in MCP-1 secretion in 3T3-L1 cells (p < 0.05, Fig 2A). In the other inflammatory model, 3T3-L1 cells were cultured in RAW-CM in order to stimulate inflammation in adipocytes (Fig 2B). MCP-1 cytokine secretion of cells treated with 2 and 5 mM aspirin in RAW-CM displayed significantly decreased by 41% and 64% (p < 0.001) respectively, compared with the control.

### Aspirin inhibited 4T1 breast cancer cell proliferation, migration, and cytokine secretion relative to tumor characteristics

**Fig 3:**
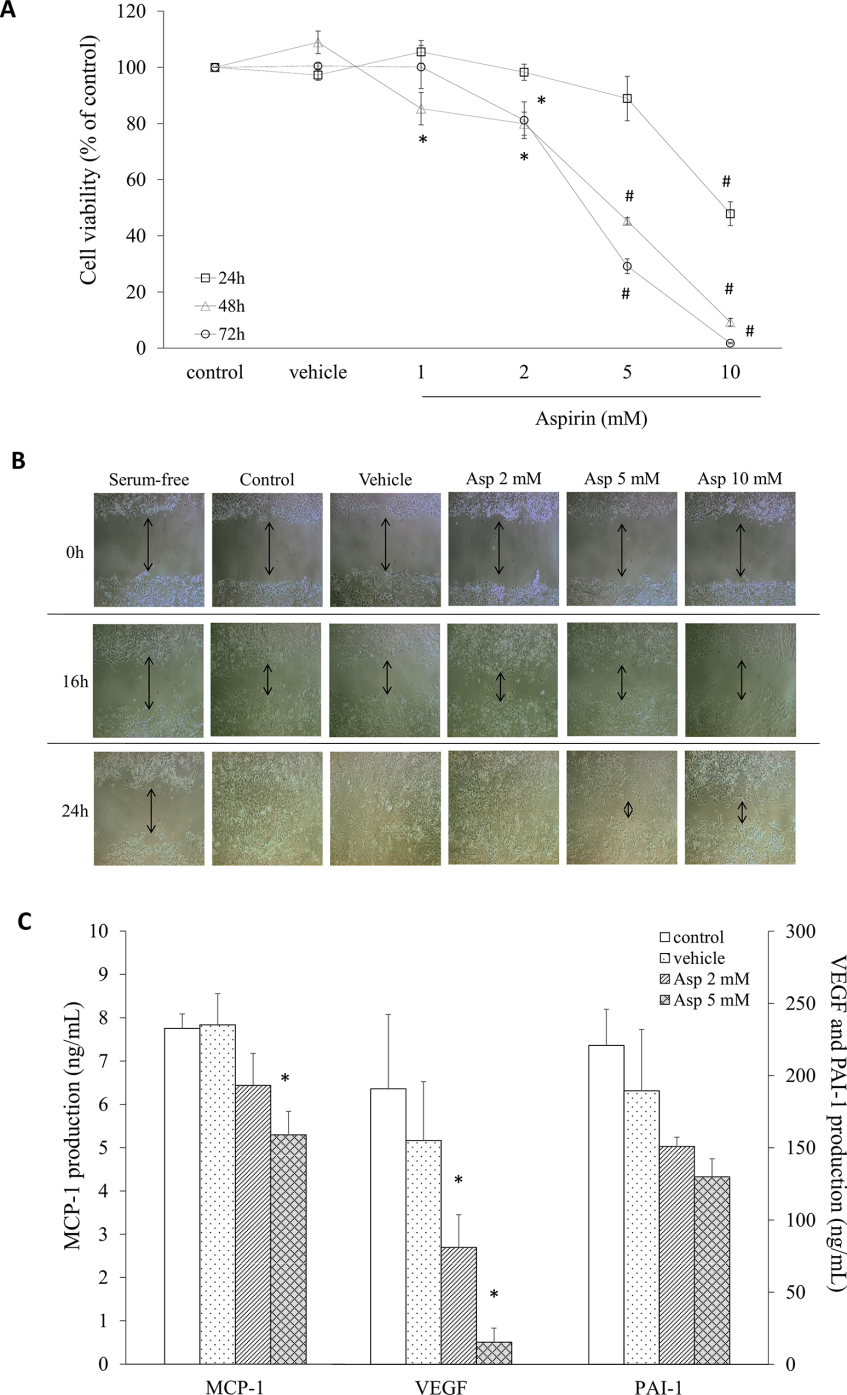
Cell growth and migration of 4T1 cells treated with aspirin. **A.** The proliferation of cells treated with or without aspirin for 24, 48, and 72 h was analyzed using an MTT test. **B.** Migration patterns into the scraped area were observed for each condition and 4T1 cells were treated with or without aspirin for 16 and 24 h. **C.** The production of cytokines characteristic of tumors in the supernatant after a 24-h culture was measured by ELISA. Data are presented as mean ± SEM. Statistical analysis was done by one-way ANOVA and LSD post hoc test; significantly different at * p < 0.05 or # p < 0.001 vs. control group.

To investigate the effect of aspirin on cell growth in cultured 4T1 cells, an MTT viability assay was performed. As shown in Fig 3A, treatment with aspirin inhibited the viability of 4T1 cells in a dose-dependent manner at 24, 48, and 72 h. When 4T1 cells were treated with 1, 2, 5, and 10 mM aspirin for 48 h, aspirin significantly inhibited the proliferation of 4T1 cells by 15%, 20%, 55% and 91%, respectively, compared with untreated cells. Cell migration was assessed using the wound-healing assay performed on a confluent monolayer of cells that was wounded by scraping with a pipette tip. Cells were serum starved as a negative control, resulting in an absence of cell proliferation after 24 h culture (Fig 3B). At 24 h post-wounding, full migration of 4T1 cells was measured in the untreated control group, whereas aspirin treatment delayed healing after scraping in a concentration-dependent manner (Fig 3B).

To investigate the effects of aspirin treatment on the cytokine/chemokine secretion characteristic of tumor cells, 4T1 cells were treated with aspirin for 24 h, and the supernatants were collected and assayed for MCP-1, VEGF, and PAI-1 (Fig 3C). Cytokine production was decreased with 2 and 5 mM aspirin treatment by about 18.1% and 40.1% (p = 0.012) for MCP-1, and 56.6% and 94.6% (p = 0.047; p = 0.004) for VEGF, respectively, compared with the control. Aspirin also decreased PAI-1 secretion by 15% and 32%, but there was no significant difference (Fig 3C).

### Aspirin inhibited breast cancer cell growth and angiogenesis cytokine secretion in 4T1 cells cultured in Ad-CM

**Fig 4:**
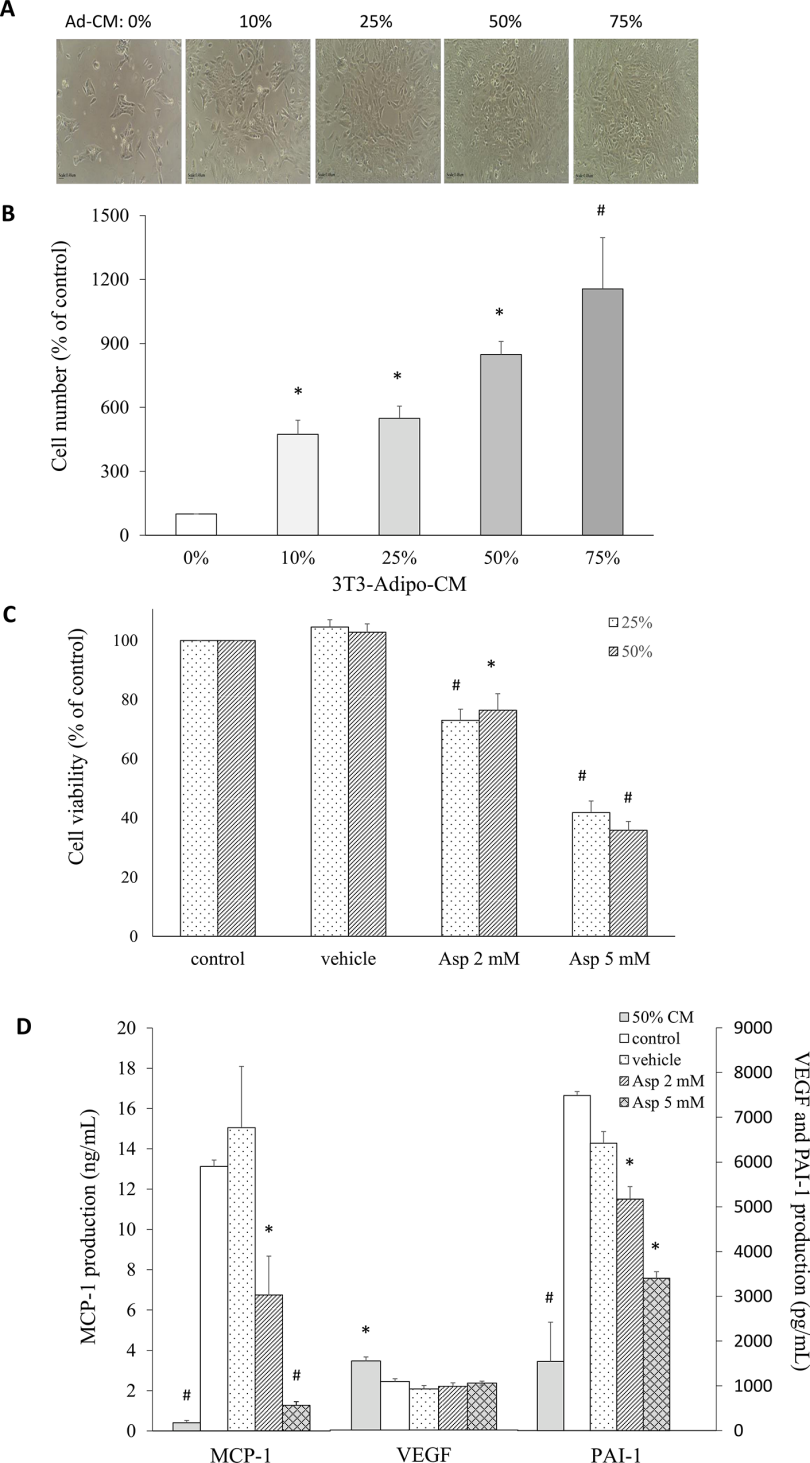
Aspirin treatment of 4T1 breast cancer cells cultured in 3T3-L1 adipocyte-conditioned medium (Ad-CM) affected the proliferative ability and cytokine production of breast cancer cells. **A.** The morphology of 4T1 cells cultured in the presence of increasing concentrations of Ad-CM for 48 h was observed microscopically at 100× magnification. **B.** Effect of the concentration of Ad-CM on the proliferation of 4T1 cells. Cells were cultured in the presence of increasing concentrations of Ad-CM for 48 h, and were then trypsinized and counted by NC-3000. **C.** The 4T1 cells were cultured either in 25% or 50% Ad-CM with or without aspirin for 48 h, and a cell proliferation assay was performed. **D.** The production of characteristic tumor cytokines after 48-h culture was measured in the supernatant by ELISA. Data are presented as mean ± SEM. Statistical analysis was done by one-way ANOVA and LSD post hoc test; significantly different at * p < 0.05 or # p < 0.001 vs. control group.

To determine the effect of Ad-CM on breast cancer cell growth, different concentrations of Ad-CM were added to the culture medium of 4T1 cells. The 4T1 cells were cultured for 48 h in 10%, 25%, 50%, and 75% Ad-CM in fresh complete medium. The group was defined as 0% Ad-CM, which means cells were cultured without conditioned medium, and this showed the least number of cells. A progressive increase in the number in 4T1 cells was observed compared with the number in the control group, and this depended on the concentration of Ad-CM. For 10%, 25%, 50%, and 75% Ad-CM, the number of cells significantly increased to 474%, 548%, 848%, and 1156%, respectively, compared with cells cultured without CM (Fig 4A and 4B). After 2 and 5 mM aspirin treatment, the number of live 4T1 cells was significantly decreased for both the 25% and 50% Ad-CM concentrations (p < 0.001 and p = 0.001, respectively) (Fig 4C). To evaluate the effect of aspirin on cytokine/chemokine secretion, 4T1 cells were cultured in 50% Ad-CM. The levels of MCP-1, VEGF, and PAI-1 secreted by 4T1 cells were measured by ELISA (Fig 4D). MCP-1 and PAI-1 levels were significantly lower in the 50% Ad-CM medium in the absence of 4T1 cells, suggesting breast cancer cells were stimulated by Ad-CM to secrete these cytokines, and adipose cells might support breast cancer development. Treatment of 4T1 cells with 2 and 5 mM aspirin resulted in significant decreases in MCP-1 secretion by about 49% and 90% (p = 0.019 and p < 0.001, respectively) and PAI-1 secretion by about 31% and 55% (p = 0.013 and p = 0.011, respectively) compared with the control group, but there was no effect on VEGF production.

### Aspirin breaks the cross talk between breast cancer cells and adipocytes

**Fig 5:**
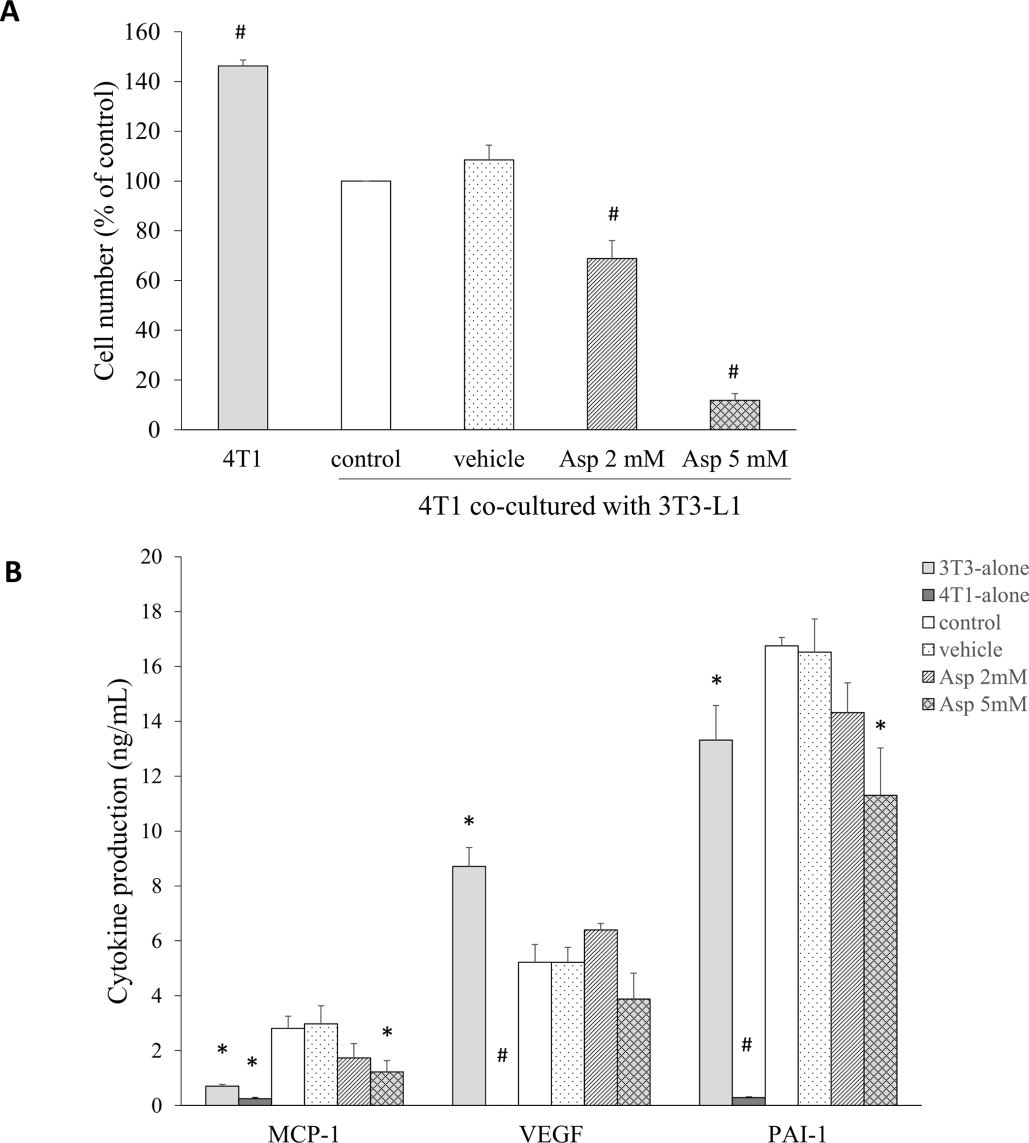
Aspirin treatment in a coculture of 4T1 breast cancer cells and mature 3T3-L1 adipocytes affected the proliferative ability and cytokine production of breast cancer cells. **A.** The 4T1 cells were cultured for 48 h in the presence or absence of mature adipocytes using a transwell system. Cells were trypsinized and cell numbers counted by NC-3000. **B.** The secretion of cytokines relevant to tumor characteristics in the supernatant obtained from 48 h coculture was measured by ELISA. Data are presented as mean ± SEM. Statistical analysis was done by one-way ANOVA and LSD post hoc test; significantly different at * p < 0.05 or # p < 0.001 vs. control group.

Potential mediators of the interactions between 3T3-L1 adipocytes and 4T1 breast cancer cells were evaluated using a transwell co-culture system. After 2 and 5 mM aspirin treatment for 48 h, the number of live 4T1 cells was significant decreased by 31% and 88% (p < 0.001), compared with the control group (Fig 5A). At the end of the co-culture period, the cytokines MCP-1, VEGF, and PAI-1 in the culture media were measured by ELISA (Fig 5B). The levels of MCP-1, VEGF, and PAI-1 in the medium with only 4T1 cells present were very low. When both cell types were present, the MCP-1 production was inhibited by treatment with 2 and 5 mM aspirin by approximately 38% and 57% (p = 0.098 and p = 0.022, respectively), and there was a 15% and 33% decrease in PAI-1 secretion (p = 0.145 and p = 0.005, respectively). This indicates that aspirin treatment blocked the production of mediators relevant to inflammatory and angiogenic processes in the co-culture system, and might significantly contribute to the suppression of obesity-related breast cancer development.

## Discussion

Obesity has been associated with an increase in the incidence and mortality of various kinds of cancer, including breast cancer [5,6]. The infiltration of immune cells into adipose tissue creates a low-grade inflammatory environment, and might trigger cancer development [2–4]. Accumulating evidence has shown that regular use of aspirin results in an apparent reduction in the incidence and mortality of cancer [13,16,17]. Here, we investigated the role of aspirin against inflammation and cellular growth in 3T3-L1 adipocytes and 4T1 breast cancer cells and then explored the crosstalk between these two types of cells. This study highlights the chemopreventive properties of aspirin linked the models of obesity and breast cancer. The results have indicated that aspirin treatment inhibits the differentiation of 3T3-L1 adipocytes and the secretion of the inflammatory adipokine MCP-1. In 4T1 cells, treatment with aspirin decreased MCP-1 and VEGF production, and inhibited cell viability and migration. Subsequently, the co-culture system was established to clarify the relationship between these two cell types. Aspirin treatment significantly inhibited the proliferation of 4T1 cells. This effect was associated with a decrease in MCP-1 and PAI-1 production in both the Ad-CM model and the transwell system. This study is the first to reveal that aspirin can not only inhibit the growth of 4T1 breast cancer cells, but also block the crosstalk between breast cancer cells and adipocytes *in vitro*, thereby suppressing the growth of breast cancer cells in an obesity-related environment.

Adipocytes release free fatty acids and nutrients that can be taken up by tumor cells as well as other non-adipose cells, resulting in metabolic dysfunction [21]. It has been demonstrated that the treatment of adipocytes with aspirin during maturation results in a significant decrease in the accumulation of triacylglycerol and fats [22]. Recently, Su and coworkers also showed that aspirin significantly inhibited adipose accumulation in preadipocytes through inhibition of p53-dependent pathways and this was associated with the inactivation of the pentose phosphate pathway [23]. The results of this study are consistent with previous studies in which aspirin treatment inhibited 3T3-L1 preadipocyte differentiation and fat accumulation in a dose-dependent manner [22,23].

The microenvironment of adipose tissue brings about a progressive increase in the infiltration of various types of immune cells including macrophages and lymphocytes, due to the secretion of pro-inflammatory cytokines involved in both obesity and cancer development [2,3,12]. The presence of multiple cytokines triggers inflammatory signaling and the stress response, leading to insulin resistance through the activation of JNK1 and IKK pathways [21], thereby supporting tumor progression and uncontrolled growth [24]. In 3T3-L1 preadipocytes, administration of LPS and RAW-CM both increased IL-6 and TNF-α production and IL-6 gene expression, but decreased levels of the PPARγ gene [25]. However, aspirin treatment of 3T3-L1 preadipocytes cultured in RAW-CM dampened the proinflammatory responses [25], by suppressing the phosphorylation of NF-κB, thus down-regulating inflammatory adipokines [26]. This suppressive effect has also been shown in both human and mouse studies, in which the production of IL-6 in the serum of obese women was significantly decreased after taking aspirin for 10 days, and the level of IL-6 in the white adipose tissue of mice was also lower after aspirin treatment [27]. Therefore, stimulation by TNF-α and RAW-CM was carried out to evaluate aspirin’s anti-inflammation properties on 3T3-L1 adipocytes in this study. These results were consistent with the findings of other researchers, and aspirin contributed to lower fat accumulation in adipocytes and adipokine secretion of signaling molecules such as MCP-1.

MCP-1, also known as CCL2, is one of chemokine superfamily that plays a crucial role in the recruitment and activation of monocytes during acute inflammation and angiogenesis. In obese subjects, MCP-1 and IL-6 levels in the circulation are frequently higher than those in thin animals [2]. It has been demonstrated that the adipokines MCP-1 and IL-6 indirectly promote tumorigenesis through infiltrative macrophage inflammation leading to cancer development [28]. Moreover, tumor-associated macrophages produced IL-10 and transforming growth factor-β, which suppressed Th1 lymphocytes, and recruited a Th2 response and regulatory T cells, promoting angiogenesis, wound healing, and pro-tumoral effectors [28]. This indicates that lower levels of MCP-1 and IL-6 might benefit and enhance the immune response of subjects against neoplastic diseases. Recently, Song and coworker have demonstrated that 4T1 cell conditioned medium stimulated mice monocyte migration, which was suppressed by a CCR2 (CCL2 receptor) antibody referred to block CCL2/CCR2-signaling [29]. In the other 4T1 cells implanted tumor model, an anti-CCL2 monoclonal antibody was administered intraperitoneally, resulting inhibit the growth of primary malignant lesions and reduce the number of pulmonary metastatic spread in BALB/c mice [30]. Recently, several studies have shown that neutralization of MCP-1 reduced metastasis and improved survival in mice tumorigenic models [31], proving that MCP-1 is a mediator associated to tumors growth and metastasis. However, long-term MCP-1 neutralization is associated the consequences of compromised immunosurveillance need to consider deeply [31].

Cancer development elevates the production of these inflammatory mediators, which may serve as potential biomarkers of cancer malignancy, suggesting that inhibiting some of these mediators could be a novel approach against cancer [32]. Many immune cells infiltrate the microenvironment of tumor and adipose tissue, thus promoting the survival of neoplastic cells by producing several growth factors and cytokines. Therefore, in the obesity-related cancer environment where many cytokines show altered profiles, angiogenesis factors (i.e., EGF, VEGF, and PAI-1) and inflammatory mediators (i.e., IL-1, IL-6, TNF-α, and leptin) are thought to cultivate a favorable environment for neoplastic cell growth. It has been demonstrated that the level of VEGF is elevated in the circulation of obese people, and is accompanied by tumor expression, which is associated with a poor prognosis in obesity-related cancers [12,33]. Recently, it was revealed that the levels of VEGF and TNF-α in serum of tumor bearing mice were higher than the control group after implanting 4T1 cells for 10 days, but the CCL2 level was increased quickly after implantation [34]. It is clear that these inflammatory mediators are a response to breast tumor growth. Moreover, 4T1 cells mediated the augmentation of active macrophage by LPS, and increased phagocytosis and CCL2 secretion [35], suggesting that 4T1 cells enhanced macrophage-mediated inflammatory responses. Our data have demonstrated that aspirin significantly inhibited the production of inflammatory and angiogenic mediators such as MCP-1 and VEGF, suppressing 4T1 cells growth and migration.

A compelling body of epidemiologic and experimental evidence has revealed the chemopreventive properties of aspirin. Observational studies have revealed that the regular use of aspirin reduces several cancers incidence and their distant metastases [36]. Aspirin use in women diagnosed with breast cancer has been associated with a decreased risk of distant recurrence and cancer death [16]. Another study has indicated an effective role for aspirin in digestive tract cancers, with modest risk reductions also being observed for breast and prostate cancers [17]. Scientific evidence supports aspirin as one of the strongest drugs, reducing breast cancer mortality by 50% [37]. To date, several meta-analyses have supported the chemopreventive effects of aspirin or NSAIDs against breast cancer [38,39]. On the basis of research findings, regular use of aspirin results in an apparent reduction in breast cancer incidence and mortality in observational studies, but the effects are not yet unequivocal. The dosage of aspirin range from 75 to 350 mg/day was used to lower cancers risk in epidemiologic experiments has been reported [37–40]. Aspirin is prone to undesirable side effects, in particular ulceration and bleeding of the gastrointestinal tract. Thus, not every study supports its use for chemoprevention [40]. The concentrations of aspirin required for the reported effect are high, thus the clinical relevance and application are questionable. More formal studies are needed to precisely determine the benefits and risks of aspirin use, such as optimal dosage and duration, and the types of cancer that respond to aspirin. In addition, developing a safer form of aspirin or a combination drug should be considered urgently to improve its efficacy.

A growing list of possible mechanisms linking obesity and breast cancer has been proposed, especially in postmenopausal women. However, the detailed molecular mechanisms of obesity-related breast tumorigenesis have not been thoroughly explored. Accumulating evidence has shown that obesity promotes mammary tumor growth and development in obese animal models [6]. Both dietary-induced obesity and genetic obesity promoted mammary tumorigenesis and increased mortality [41]. In obese mice, the expression of VEGF, IKKβ, and mTOR are up-regulated in tumors [41] and the levels of various adipokines are modulated during the development of obesity, perhaps crucially contributing to breast carcinogenesis [42]. *In vitro*, treatment of MCF-7 and MDA-MB-231 human breast cancer cells with leptin enhanced VEGF secretion, and increased cell growth and migration compared with the no-treatment condition, whereas adiponectin treatment showed the reverse effects [42], suggesting that obesity indirectly promotes breast tumorigenesis. In this study, we also determined the chemopreventive properties of aspirin in this complex environment of obesity-related breast cancer. Treatment with aspirin significantly decreased the number of 4T1 cells in both experimental systems, and might inhibit MCP-1 and PAI-1 secretion in the cultured supernatant. PAI-1 is frequently shown to be higher in the serum of obese subjects, and decreases cell binding to extracellular matrix proteins, thus promoting tumor cell detachment from tissues [12]. Moreover, PAI-1 has been shown to promote tumor growth and inhibit apoptosis, and is an indicator of poor prognosis in breast cancer [43], implying that lower PAI-1 levels might control the growth of breast cancer cells in this study.

**Fig 6:**
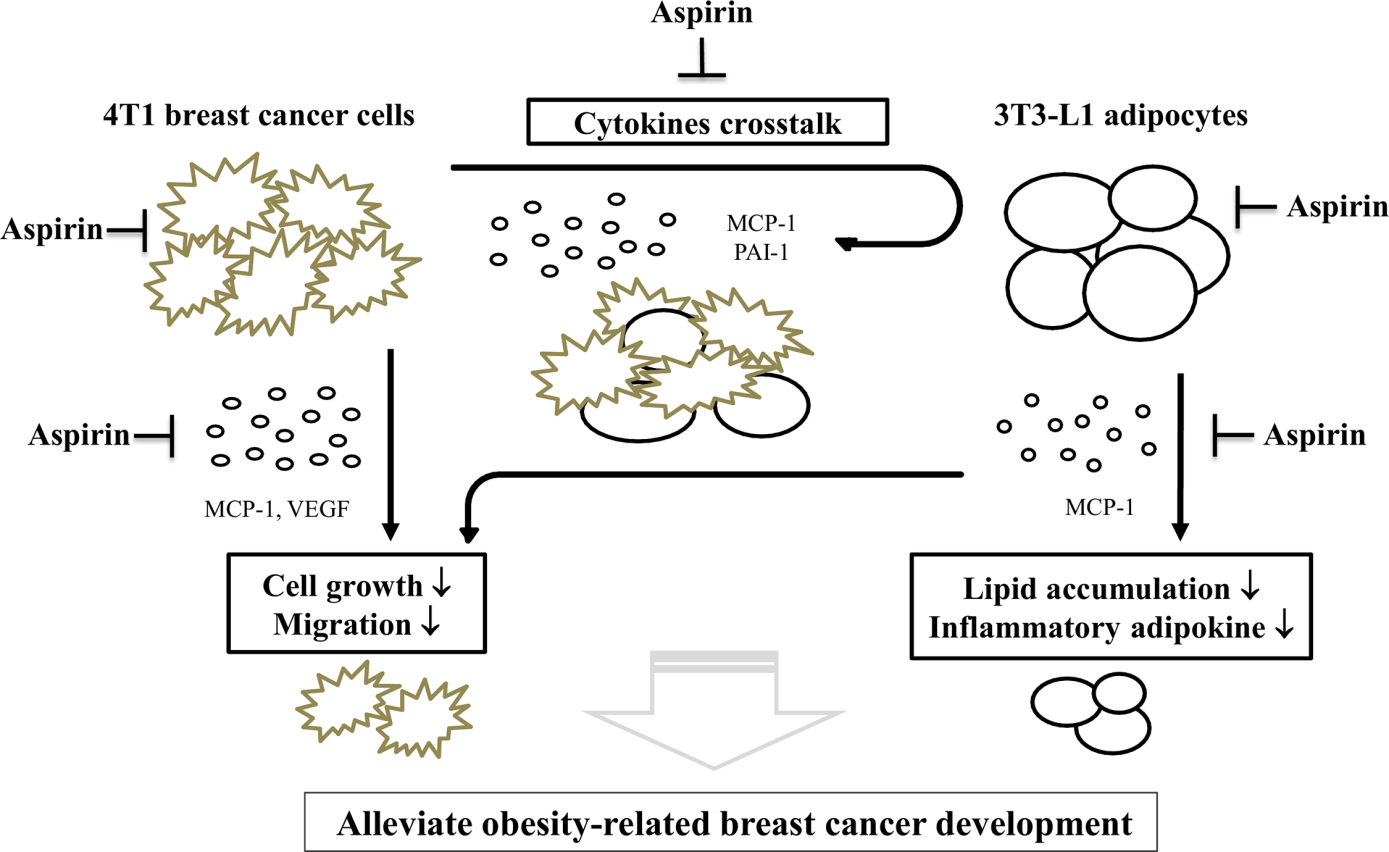
The proposed schema of the mechanisms by which aspirin breaks the crosstalk between breast cancer cells and adipocytes. In 3T3-L1 cells, aspirin treatment inhibited the differentiation of adipocytes and the secretion of the inflammatory adipokine MCP-1. In 4T1 cells, treatment with aspirin decreased MCP-1 and VEGF production, and inhibited cell viability and migration. Subsequently, to clarify the relationship between these two cell types by the co-culture systems, including Ad-CM model and transwell system. Aspirin treatment significantly inhibited the proliferation of 4T1 cells, possibly by inhibiting MCP-1 and PAI-1 secretion and further diminishing the proliferation and migration of 4T1 cells. The arrows denote changes that display due to aspirin treatment.

In summary, the tentative schema is proposed for the possible mechanisms of action of aspirin in this study (Fig 6). Breast cancer progression and metastasis are dependent on the secretion, actions, and crosstalk among a series of cytokines. However, there is still little evidence on the mechanisms relevant to obesity-mediated breast carcinogenesis. This study has provided the first findings showing that aspirin is effective not only against 4T1 breast cancer cells, but can also break the crosstalk between breast cancer cells and adipocytes, possibly by inhibiting MCP-1 and PAI-1 secretion and further diminishing the proliferation and migration of 4T1 cells. Based on the finding that aspirin use might inhibit inflammatory responses and angiogenesis in obesity-related breast cancer development, taking aspirin might control the course of breast tumorigenesis. In addition, aspirin use in these obese subjects could improve their cardiovascular health and reduce the risk of heart attack. Interestingly, in obese subjects, aspirin use could provide a considerable improvement in auxiliary prevention and therapy.
